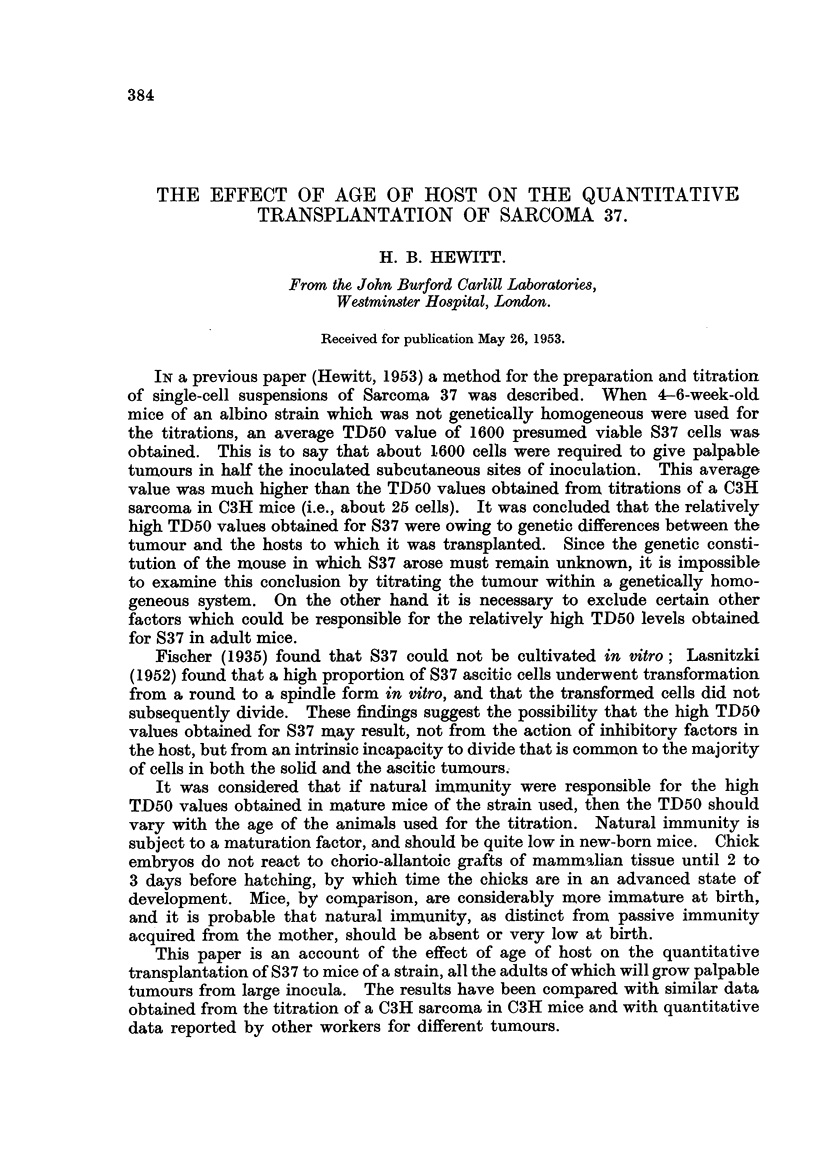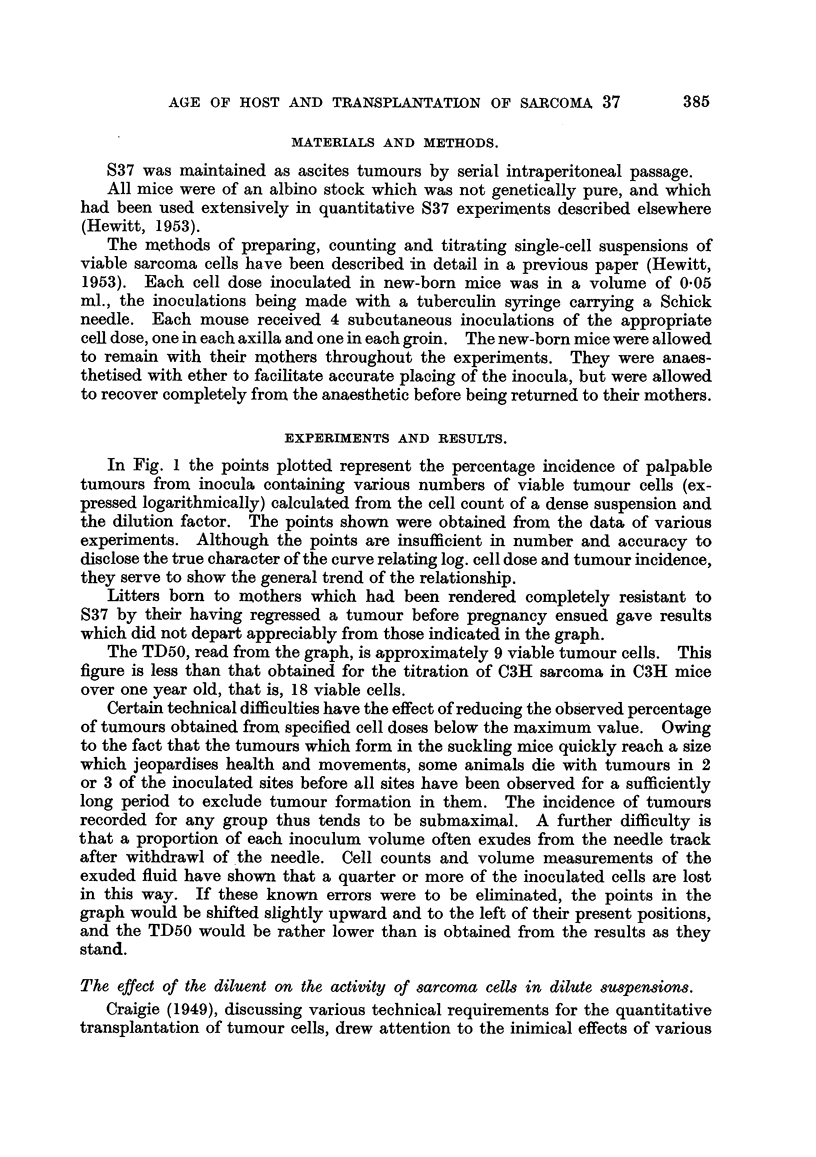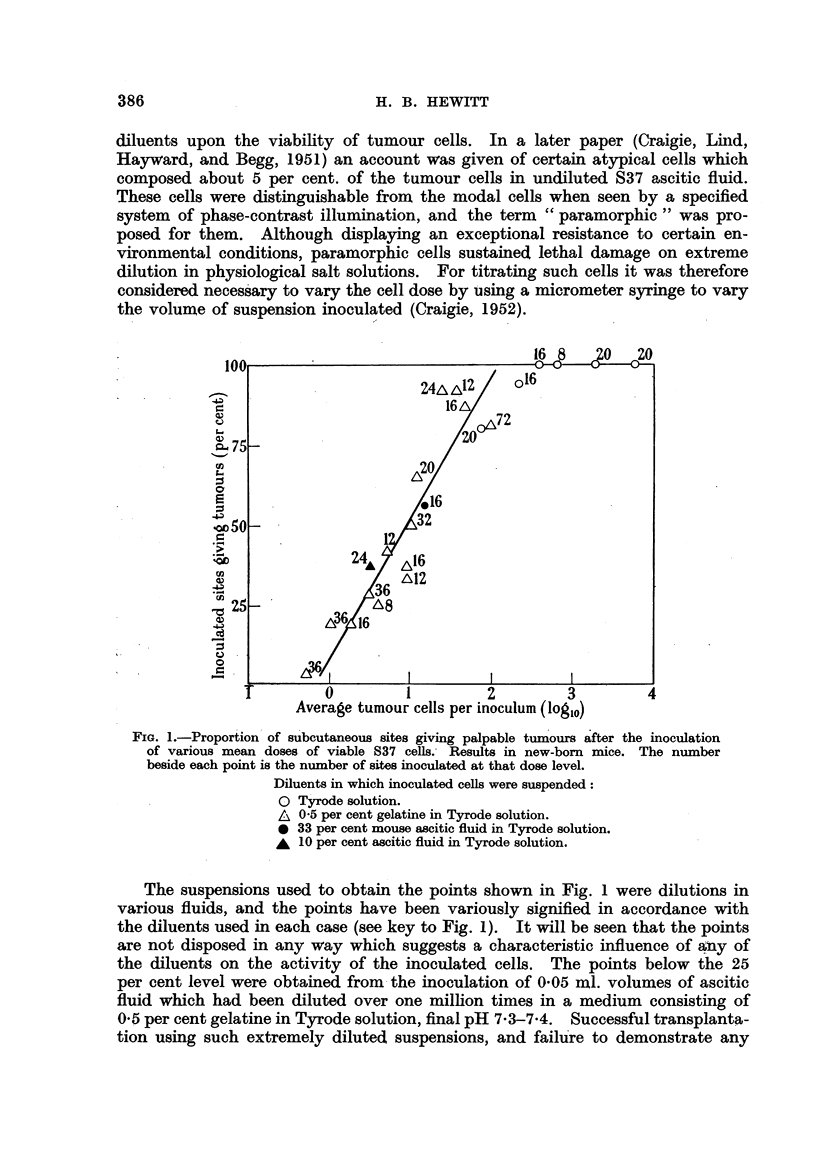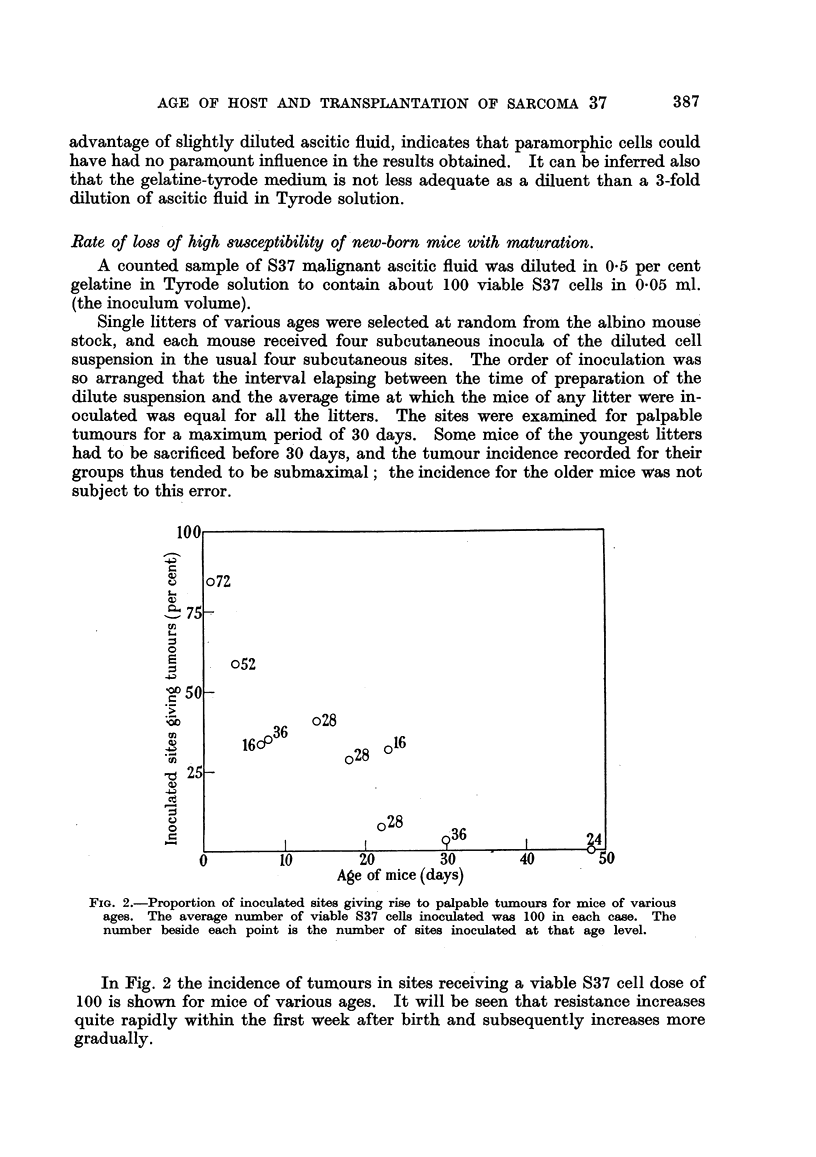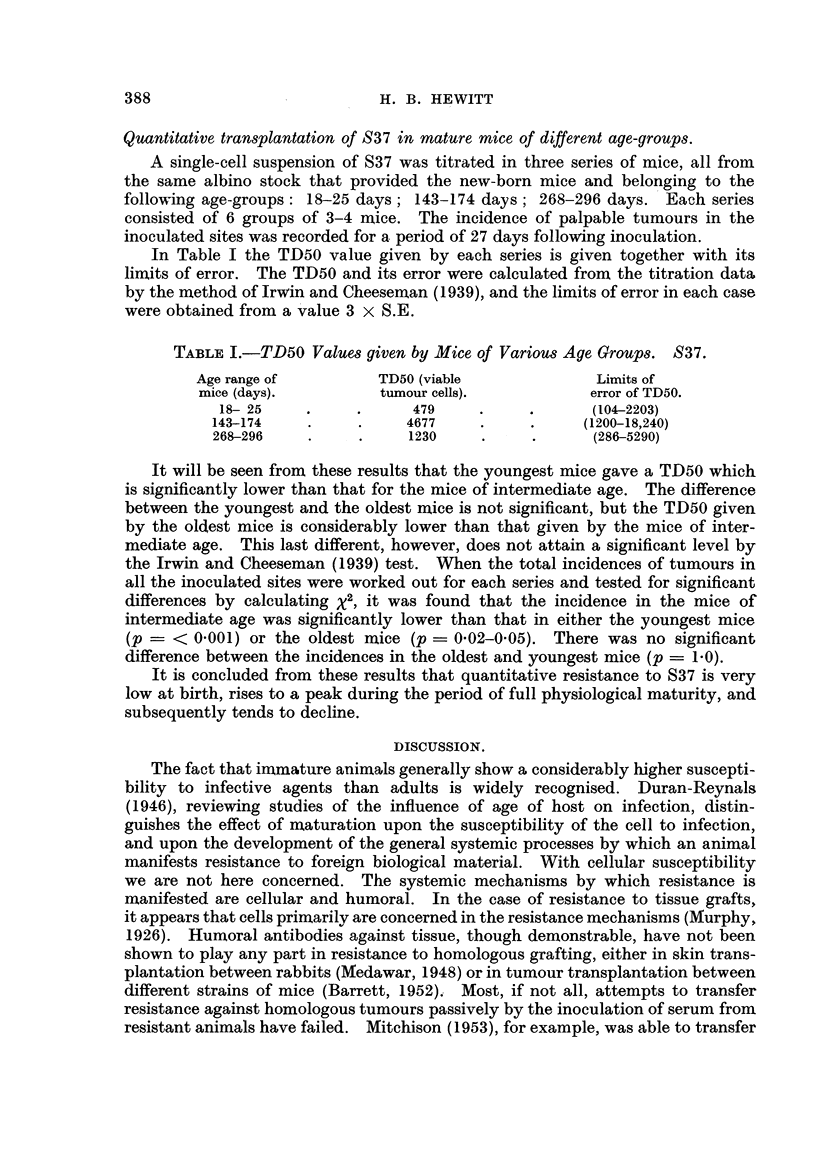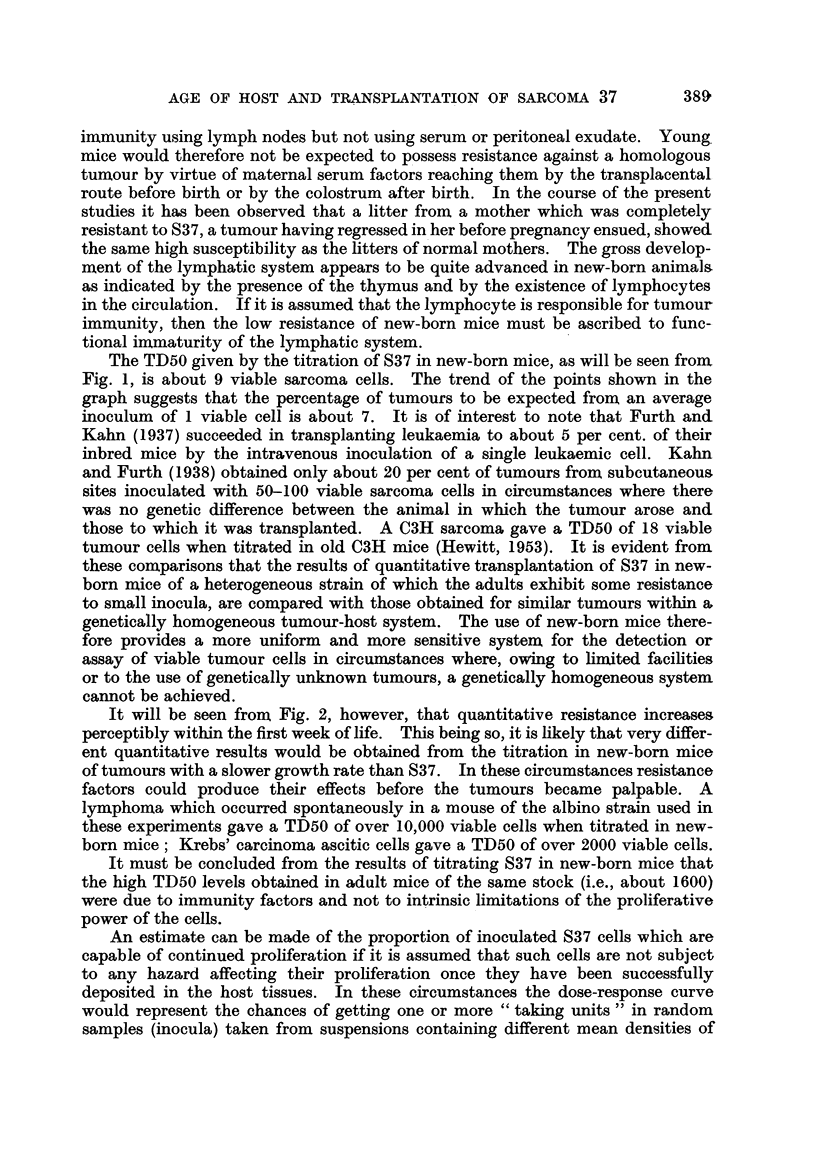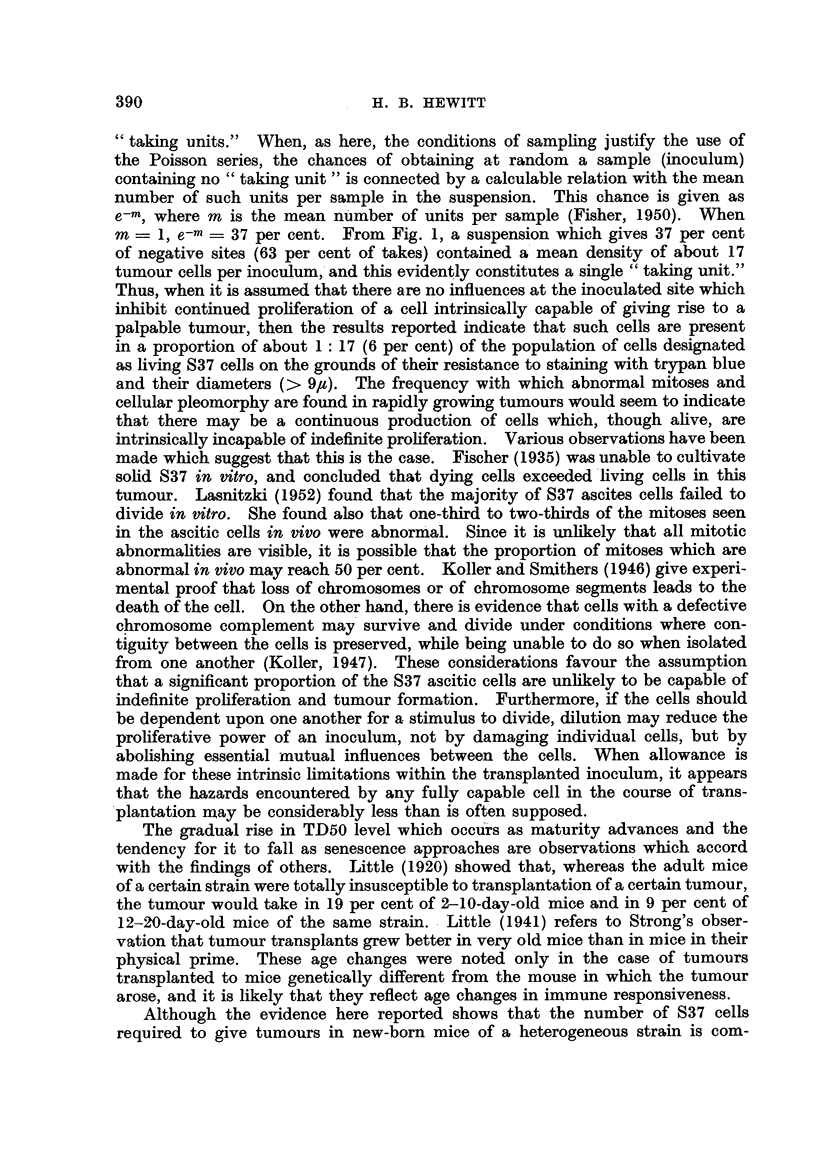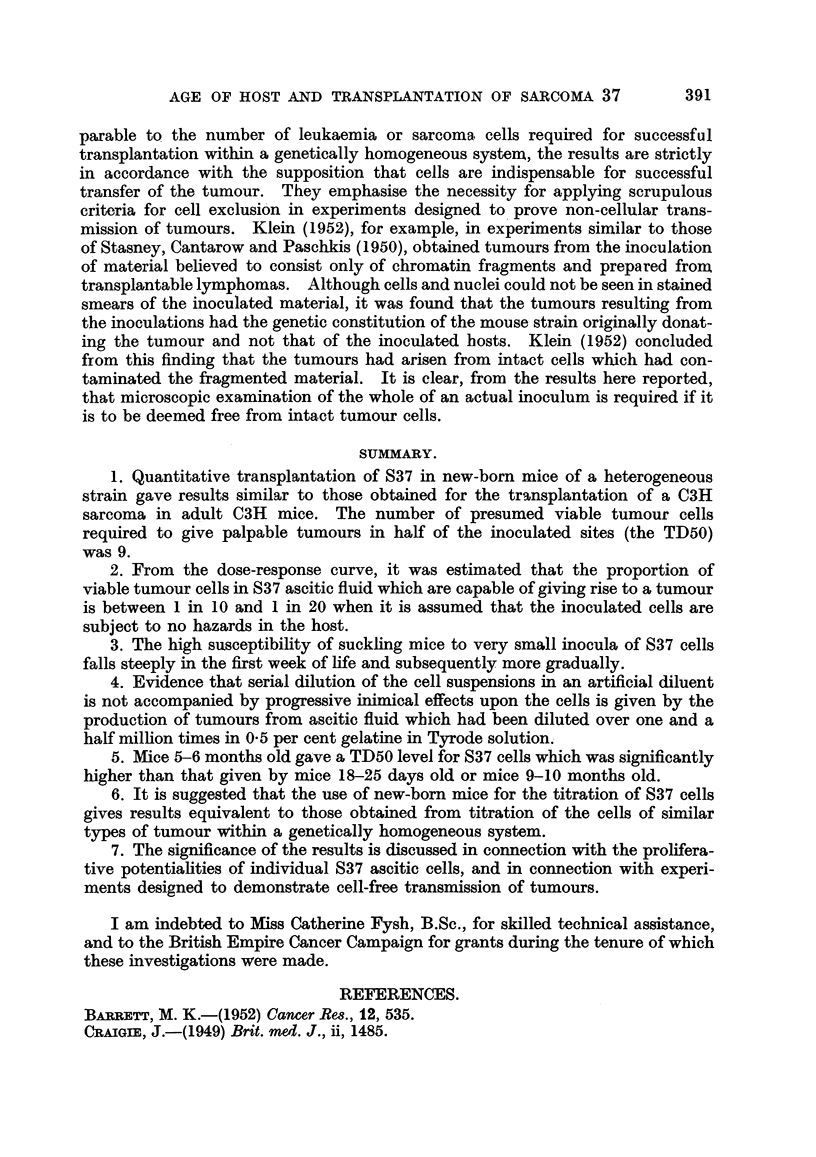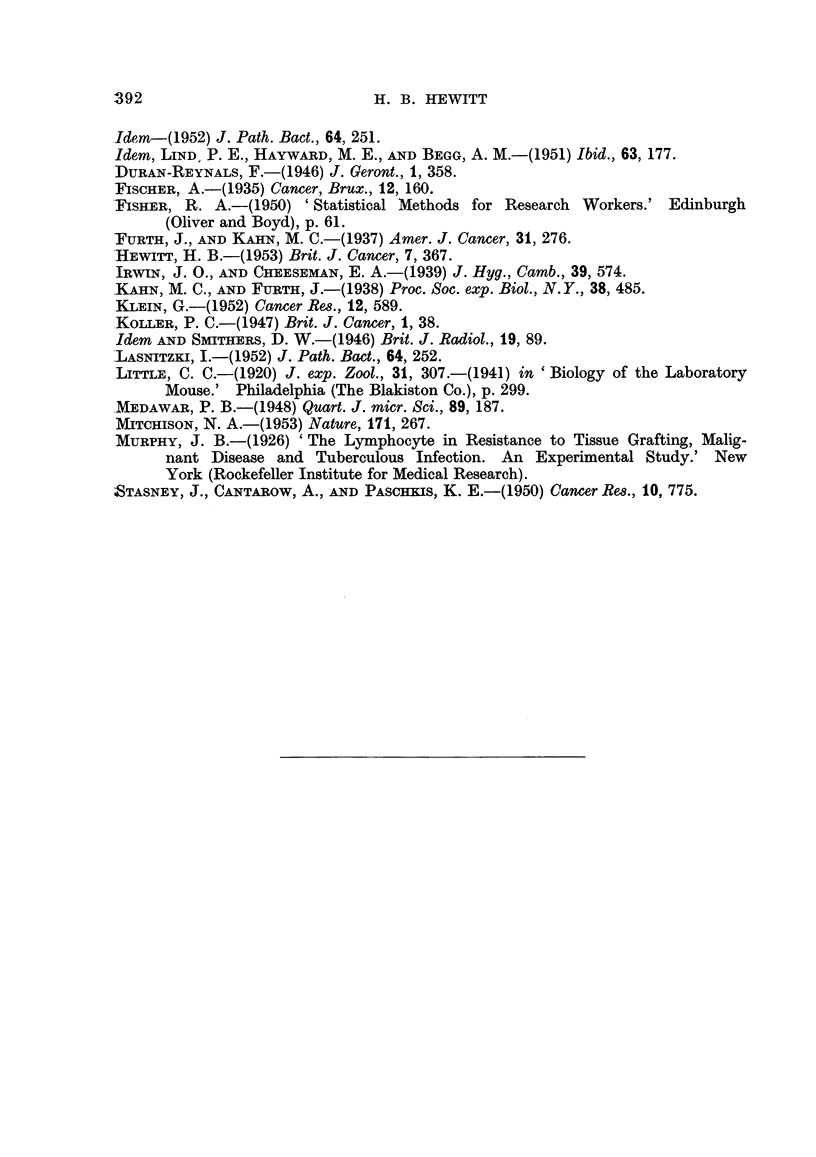# The Effect of Age of Host on the Quantitative Transplantation of Sarcoma 37

**DOI:** 10.1038/bjc.1953.36

**Published:** 1953-09

**Authors:** H. B. Hewitt


### THE EFFECT OF AGE OF HOST ON THE QUANTITATIVE TRANSPLANTATION OF SARCOMA 37.

H. B. HEWITT.

*From the John Burford Carlill Laboratories, Westminster Hospital, London.*

Received for publication May 26, 1953.

IN a previous paper (Hewitt, 1953) a method for the preparation and titration of single-cell suspensions of Sarcoma 37 was described. When 4–6-week-old mice of an albino strain which was not genetically homogeneous were used for the titrations, an average TD50 value of 1600 presumed viable S37 cells was obtained. This is to say that about 1600 cells were required to give palpable tumours in half the inoculated subcutaneous sites of inoculation. This average value was much higher than the TD50 values obtained from titrations of a C3H sarcoma in C3H mice (i.e., about 25 cells). It was concluded that the relatively high TD50 values obtained for S37 were owing to genetic differences between the tumour and the hosts to which it was transplanted. Since the genetic constitution of the mouse in which S37 arose must remain unknown, it is impossible to examine this conclusion by titrating the tumour within a genetically homogeneous system. On the other hand it is necessary to exclude certain other factors which could be responsible for the relatively high TD50 levels obtained for S37 in adult mice.

Fischer (1935) found that S37 could not be cultivated *in vitro*; Lasnitzki (1952) found that a high proportion of S37 ascitic cells underwent transformation from a round to a spindle form *in vitro*, and that the transformed cells did not subsequently divide. These findings suggest the possibility that the high TD50 values obtained for S37 may result, not from the action of inhibitory factors in the host, but from an intrinsic incapacity to divide that is common to the majority of cells in both the solid and the ascitic tumours.

It was considered that if natural immunity were responsible for the high TD50 values obtained in mature mice of the strain used, then the TD50 should vary with the age of the animals used for the titration. Natural immunity is subject to a maturation factor, and should be quite low in new-born mice. Chick embryos do not react to chorio-allantoic grafts of mammalian tissue until 2 to 3 days before hatching, by which time the chicks are in an advanced state of development. Mice, by comparison, are considerably more immature at birth, and it is probable that natural immunity, as distinct from passive immunity acquired from the mother, should be absent or very low at birth.

This paper is an account of the effect of age of host on the quantitative transplantation of S37 to mice of a strain, all the adults of which will grow palpable tumours from large inocula. The results have been compared with similar data obtained from the titration of a C3H sarcoma in C3H mice and with quantitative data reported by other workers for different tumours.

### MATERIALS AND METHODS.

S37 was maintained as ascites tumours by serial intraperitoneal passage.

All mice were of an albino stock which was not genetically pure, and which had been used extensively in quantitative S37 experiments described elsewhere (Hewitt, 1953).

The methods of preparing, counting and titrating single-cell suspensions of viable sarcoma cells have been described in detail in a previous paper (Hewitt, 1953). Each cell dose inoculated in new-born mice was in a volume of 0·05 ml., the inoculations being made with a tuberculin syringe carrying a Schick needle. Each mouse received 4 subcutaneous inoculations of the appropriate cell dose, one in each axilla and one in each groin. The new-born mice were allowed to remain with their mothers throughout the experiments. They were anaesthetised with ether to facilitate accurate placing of the inocula, but were allowed to recover completely from the anaesthetic before being returned to their mothers.

### EXPERIMENTS AND RESULTS.

In Fig. 1 the points plotted represent the percentage incidence of palpable tumours from inocula containing various numbers of viable tumour cells (expressed logarithmically) calculated from the cell count of a dense suspension and the dilution factor. The points shown were obtained from the data of various experiments. Although the points are insufficient in number and accuracy to disclose the true character of the curve relating log. cell dose and tumour incidence, they serve to show the general trend of the relationship.

Litters born to mothers which had been rendered completely resistant to S37 by their having regressed a tumour before pregnancy ensued gave results which did not depart appreciably from those indicated in the graph.

The TD50, read from the graph, is approximately 9 viable tumour cells. This figure is less than that obtained for the titration of C3H sarcoma in C3H mice over one year old, that is, 18 viable cells.

Certain technical difficulties have the effect of reducing the observed percentage of tumours obtained from specified cell doses below the maximum value. Owing to the fact that the tumours which form in the suckling mice quickly reach a size which jeopardises health and movements, some animals die with tumours in 2 or 3 of the inoculated sites before all sites have been observed for a sufficiently long period to exclude tumour formation in them. The incidence of tumours recorded for any group thus tends to be submaximal. A further difficulty is that a proportion of each inoculum volume often exudes from the needle track after withdrawl of the needle. Cell counts and volume measurements of the exuded fluid have shown that a quarter or more of the inoculated cells are lost in this way. If these known errors were to be eliminated, the points in the graph would be shifted slightly upward and to the left of their present positions, and the TD50 would be rather lower than is obtained from the results as they stand.

*The effect of the diluent on the activity of sarcoma cells in dilute suspensions.*

Craigie (1949), discussing various technical requirements for the quantitative transplantation of tumour cells, drew attention to the inimical effects of various diluents upon the viability of tumour cells. In a later paper (Craigie, Lind, Hayward, and Begg, 1951) an account was given of certain atypical cells which composed about 5 per cent. of the tumour cells in undiluted S37 ascitic fluid. These cells were distinguishable from the modal cells when seen by a specified system of phase-contrast illumination, and the term "paramorphic" was proposed for them. Although displaying an exceptional resistance to certain environmental conditions, paramorphic cells sustained lethal damage on extreme dilution in physiological salt solutions. For titrating such cells it was therefore considered necessary to vary the cell dose by using a micrometer syringe to vary the volume of suspension inoculated (Craigie, 1952).

Fig. 1.—Proportion of subcutaneous sites giving palpable tumours after the inoculation of various mean doses of viable S37 cells. Results in new-born mice. The number beside each point is the number of sites inoculated at that dose level.

Diluents in which inoculated cells were suspended :
○ Tyrode solution.
△ 0·5 per cent gelatine in Tyrode solution.
● 33 per cent mouse ascitic fluid in Tyrode solution.
▲ 10 per cent ascitic fluid in Tyrode solution.

The suspensions used to obtain the points shown in Fig. 1 were dilutions in various fluids, and the points have been variously signified in accordance with the diluents used in each case (see key to Fig. 1). It will be seen that the points are not disposed in any way which suggests a characteristic influence of any of the diluents on the activity of the inoculated cells. The points below the 25 per cent level were obtained from the inoculation of 0·05 ml. volumes of ascitic fluid which had been diluted over one million times in a medium consisting of 0·5 per cent gelatine in Tyrode solution, final pH 7·3–7·4. Successful transplantation using such extremely diluted suspensions, and failure to demonstrate any advantage of slightly diluted ascitic fluid, indicates that paramorphic cells could have had no paramount influence in the results obtained. It can be inferred also that the gelatine-tyrode medium is not less adequate as a diluent than a 3-fold dilution of ascitic fluid in Tyrode solution.

*Rate of loss of high susceptibility of new-born mice with maturation.*

A counted sample of S37 malignant ascitic fluid was diluted in 0·5 per cent gelatine in Tyrode solution to contain about 100 viable S37 cells in 0·05 ml. (the inoculum volume).

Single litters of various ages were selected at random from the albino mouse stock, and each mouse received four subcutaneous inocula of the diluted cell suspension in the usual four subcutaneous sites. The order of inoculation was so arranged that the interval elapsing between the time of preparation of the dilute suspension and the average time at which the mice of any litter were inoculated was equal for all the litters. The sites were examined for palpable tumours for a maximum period of 30 days. Some mice of the youngest litters had to be sacrificed before 30 days, and the tumour incidence recorded for their groups thus tended to be submaximal; the incidence for the older mice was not subject to this error.

FIG. 2.—Proportion of inoculated sites giving rise to palpable tumours for mice of various ages. The average number of viable S37 cells inoculated was 100 in each case. The number beside each point is the number of sites inoculated at that age level.

In Fig. 2 the incidence of tumours in sites receiving a viable S37 cell dose of 100 is shown for mice of various ages. It will be seen that resistance increases quite rapidly within the first week after birth and subsequently increases more gradually.

*Quantitative transplantation of S37 in mature mice of different age-groups.*

A single-cell suspension of S37 was titrated in three series of mice, all from the same albino stock that provided the new-born mice and belonging to the following age-groups : 18–25 days ; 143–174 days ; 268–296 days. Each series consisted of 6 groups of 3–4 mice. The incidence of palpable tumours in the inoculated sites was recorded for a period of 27 days following inoculation.

In Table I the TD50 value given by each series is given together with its limits of error. The TD50 and its error were calculated from the titration data by the method of Irwin and Cheeseman (1939), and the limits of error in each case were obtained from a value 3 $\times$ S.E.

TABLE I.—*TD50 Values given by Mice of Various Age Groups. S37.*

| Age range of mice (days). | TD50 (viable tumour cells). | Limits of error of TD50. |
|---|---|---|
| 18– 25 | 479 | (104–2203) |
| 143–174 | 4677 | (1200–18,240) |
| 268–296 | 1230 | (286–5290) |

It will be seen from these results that the youngest mice gave a TD50 which is significantly lower than that for the mice of intermediate age. The difference between the youngest and the oldest mice is not significant, but the TD50 given by the oldest mice is considerably lower than that given by the mice of intermediate age. This last different, however, does not attain a significant level by the Irwin and Cheeseman (1939) test. When the total incidences of tumours in all the inoculated sites were worked out for each series and tested for significant differences by calculating $\chi^2$, it was found that the incidence in the mice of intermediate age was significantly lower than that in either the youngest mice ($p = < 0{\cdot}001$) or the oldest mice ($p = 0{\cdot}02$–$0{\cdot}05$). There was no significant difference between the incidences in the oldest and youngest mice ($p = 1{\cdot}0$).

It is concluded from these results that quantitative resistance to S37 is very low at birth, rises to a peak during the period of full physiological maturity, and subsequently tends to decline.

### DISCUSSION.

The fact that immature animals generally show a considerably higher susceptibility to infective agents than adults is widely recognised. Duran-Reynals (1946), reviewing studies of the influence of age of host on infection, distinguishes the effect of maturation upon the susceptibility of the cell to infection, and upon the development of the general systemic processes by which an animal manifests resistance to foreign biological material. With cellular susceptibility we are not here concerned. The systemic mechanisms by which resistance is manifested are cellular and humoral. In the case of resistance to tissue grafts, it appears that cells primarily are concerned in the resistance mechanisms (Murphy, 1926). Humoral antibodies against tissue, though demonstrable, have not been shown to play any part in resistance to homologous grafting, either in skin transplantation between rabbits (Medawar, 1948) or in tumour transplantation between different strains of mice (Barrett, 1952). Most, if not all, attempts to transfer resistance against homologous tumours passively by the inoculation of serum from resistant animals have failed. Mitchison (1953), for example, was able to transfer immunity using lymph nodes but not using serum or peritoneal exudate. Young mice would therefore not be expected to possess resistance against a homologous tumour by virtue of maternal serum factors reaching them by the transplacental route before birth or by the colostrum after birth. In the course of the present studies it has been observed that a litter from a mother which was completely resistant to S37, a tumour having regressed in her before pregnancy ensued, showed the same high susceptibility as the litters of normal mothers. The gross development of the lymphatic system appears to be quite advanced in new-born animals as indicated by the presence of the thymus and by the existence of lymphocytes in the circulation. If it is assumed that the lymphocyte is responsible for tumour immunity, then the low resistance of new-born mice must be ascribed to functional immaturity of the lymphatic system.

The TD50 given by the titration of S37 in new-born mice, as will be seen from Fig. 1, is about 9 viable sarcoma cells. The trend of the points shown in the graph suggests that the percentage of tumours to be expected from an average inoculum of 1 viable cell is about 7. It is of interest to note that Furth and Kahn (1937) succeeded in transplanting leukaemia to about 5 per cent. of their inbred mice by the intravenous inoculation of a single leukaemic cell. Kahn and Furth (1938) obtained only about 20 per cent of tumours from subcutaneous sites inoculated with 50–100 viable sarcoma cells in circumstances where there was no genetic difference between the animal in which the tumour arose and those to which it was transplanted. A C3H sarcoma gave a TD50 of 18 viable tumour cells when titrated in old C3H mice (Hewitt, 1953). It is evident from these comparisons that the results of quantitative transplantation of S37 in new-born mice of a heterogeneous strain of which the adults exhibit some resistance to small inocula, are compared with those obtained for similar tumours within a genetically homogeneous tumour-host system. The use of new-born mice therefore provides a more uniform and more sensitive system for the detection or assay of viable tumour cells in circumstances where, owing to limited facilities or to the use of genetically unknown tumours, a genetically homogeneous system cannot be achieved.

It will be seen from Fig. 2, however, that quantitative resistance increases perceptibly within the first week of life. This being so, it is likely that very different quantitative results would be obtained from the titration in new-born mice of tumours with a slower growth rate than S37. In these circumstances resistance factors could produce their effects before the tumours became palpable. A lymphoma which occurred spontaneously in a mouse of the albino strain used in these experiments gave a TD50 of over 10,000 viable cells when titrated in new-born mice ; Krebs' carcinoma ascitic cells gave a TD50 of over 2000 viable cells.

It must be concluded from the results of titrating S37 in new-born mice that the high TD50 levels obtained in adult mice of the same stock (i.e., about 1600) were due to immunity factors and not to intrinsic limitations of the proliferative power of the cells.

An estimate can be made of the proportion of inoculated S37 cells which are capable of continued proliferation if it is assumed that such cells are not subject to any hazard affecting their proliferation once they have been successfully deposited in the host tissues. In these circumstances the dose-response curve would represent the chances of getting one or more " taking units " in random samples (inocula) taken from suspensions containing different mean densities of

" taking units." When, as here, the conditions of sampling justify the use of the Poisson series, the chances of obtaining at random a sample (inoculum) containing no " taking unit " is connected by a calculable relation with the mean number of such units per sample in the suspension. This chance is given as $e^{-m}$, where $m$ is the mean number of units per sample (Fisher, 1950). When $m = 1$, $e^{-m} = 37$ per cent. From Fig. 1, a suspension which gives 37 per cent of negative sites (63 per cent of takes) contained a mean density of about 17 tumour cells per inoculum, and this evidently constitutes a single " taking unit." Thus, when it is assumed that there are no influences at the inoculated site which inhibit continued proliferation of a cell intrinsically capable of giving rise to a palpable tumour, then the results reported indicate that such cells are present in a proportion of about 1 : 17 (6 per cent) of the population of cells designated as living S37 cells on the grounds of their resistance to staining with trypan blue and their diameters ($> 9\mu$). The frequency with which abnormal mitoses and cellular pleomorphy are found in rapidly growing tumours would seem to indicate that there may be a continuous production of cells which, though alive, are intrinsically incapable of indefinite proliferation. Various observations have been made which suggest that this is the case. Fischer (1935) was unable to cultivate solid S37 *in vitro*, and concluded that dying cells exceeded living cells in this tumour. Lasnitzki (1952) found that the majority of S37 ascites cells failed to divide *in vitro*. She found also that one-third to two-thirds of the mitoses seen in the ascitic cells *in vivo* were abnormal. Since it is unlikely that all mitotic abnormalities are visible, it is possible that the proportion of mitoses which are abnormal *in vivo* may reach 50 per cent. Koller and Smithers (1946) give experimental proof that loss of chromosomes or of chromosome segments leads to the death of the cell. On the other hand, there is evidence that cells with a defective chromosome complement may survive and divide under conditions where contiguity between the cells is preserved, while being unable to do so when isolated from one another (Koller, 1947). These considerations favour the assumption that a significant proportion of the S37 ascitic cells are unlikely to be capable of indefinite proliferation and tumour formation. Furthermore, if the cells should be dependent upon one another for a stimulus to divide, dilution may reduce the proliferative power of an inoculum, not by damaging individual cells, but by abolishing essential mutual influences between the cells. When allowance is made for these intrinsic limitations within the transplanted inoculum, it appears that the hazards encountered by any fully capable cell in the course of transplantation may be considerably less than is often supposed.

The gradual rise in TD50 level which occurs as maturity advances and the tendency for it to fall as senescence approaches are observations which accord with the findings of others. Little (1920) showed that, whereas the adult mice of a certain strain were totally insusceptible to transplantation of a certain tumour, the tumour would take in 19 per cent of 2–10-day-old mice and in 9 per cent of 12–20-day-old mice of the same strain. Little (1941) refers to Strong's observation that tumour transplants grew better in very old mice than in mice in their physical prime. These age changes were noted only in the case of tumours transplanted to mice genetically different from the mouse in which the tumour arose, and it is likely that they reflect age changes in immune responsiveness.

Although the evidence here reported shows that the number of S37 cells required to give tumours in new-born mice of a heterogeneous strain is comparable to the number of leukaemia or sarcoma cells required for successful transplantation within a genetically homogeneous system, the results are strictly in accordance with the supposition that cells are indispensable for successful transfer of the tumour. They emphasise the necessity for applying scrupulous criteria for cell exclusion in experiments designed to prove non-cellular transmission of tumours. Klein (1952), for example, in experiments similar to those of Stasney, Cantarow and Paschkis (1950), obtained tumours from the inoculation of material believed to consist only of chromatin fragments and prepared from transplantable lymphomas. Although cells and nuclei could not be seen in stained smears of the inoculated material, it was found that the tumours resulting from the inoculations had the genetic constitution of the mouse strain originally donating the tumour and not that of the inoculated hosts. Klein (1952) concluded from this finding that the tumours had arisen from intact cells which had contaminated the fragmented material. It is clear, from the results here reported, that microscopic examination of the whole of an actual inoculum is required if it is to be deemed free from intact tumour cells.

### SUMMARY.

1. Quantitative transplantation of S37 in new-born mice of a heterogeneous strain gave results similar to those obtained for the transplantation of a C3H sarcoma in adult C3H mice. The number of presumed viable tumour cells required to give palpable tumours in half of the inoculated sites (the TD50) was 9.

2. From the dose-response curve, it was estimated that the proportion of viable tumour cells in S37 ascitic fluid which are capable of giving rise to a tumour is between 1 in 10 and 1 in 20 when it is assumed that the inoculated cells are subject to no hazards in the host.

3. The high susceptibility of suckling mice to very small inocula of S37 cells falls steeply in the first week of life and subsequently more gradually.

4. Evidence that serial dilution of the cell suspensions in an artificial diluent is not accompanied by progressive inimical effects upon the cells is given by the production of tumours from ascitic fluid which had been diluted over one and a half million times in 0·5 per cent gelatine in Tyrode solution.

5. Mice 5–6 months old gave a TD50 level for S37 cells which was significantly higher than that given by mice 18–25 days old or mice 9–10 months old.

6. It is suggested that the use of new-born mice for the titration of S37 cells gives results equivalent to those obtained from titration of the cells of similar types of tumour within a genetically homogeneous system.

7. The significance of the results is discussed in connection with the proliferative potentialities of individual S37 ascitic cells, and in connection with experiments designed to demonstrate cell-free transmission of tumours.

I am indebted to Miss Catherine Fysh, B.Sc., for skilled technical assistance, and to the British Empire Cancer Campaign for grants during the tenure of which these investigations were made.

### REFERENCES.

BARRETT, M. K.—(1952) *Cancer Res.*, **12**, 535.

CRAIGIE, J.—(1949) *Brit. med. J.*, ii, 1485.

*Idem*—(1952) *J. Path. Bact.*, **64**, 251.
*Idem*, LIND, P. E., HAYWARD, M. E., AND BEGG, A. M.—(1951) *Ibid.*, **63**, 177.
DURAN-REYNALS, F.—(1946) *J. Geront.*, **1**, 358.
FISCHER, A.—(1935) *Cancer, Brux.*, **12**, 160.
FISHER, R. A.—(1950) 'Statistical Methods for Research Workers.' Edinburgh (Oliver and Boyd), p. 61.
FURTH, J., AND KAHN, M. C.—(1937) *Amer. J. Cancer*, **31**, 276.
HEWITT, H. B.—(1953) *Brit. J. Cancer*, **7**, 367.
IRWIN, J. O., AND CHEESEMAN, E. A.—(1939) *J. Hyg., Camb.*, **39**, 574.
KAHN, M. C., AND FURTH, J.—(1938) *Proc. Soc. exp. Biol., N.Y.*, **38**, 485.
KLEIN, G.—(1952) *Cancer Res.*, **12**, 589.
KOLLER, P. C.—(1947) *Brit. J. Cancer*, **1**, 38.
*Idem* AND SMITHERS, D. W.—(1946) *Brit. J. Radiol.*, **19**, 89.
LASNITZKI, I.—(1952) *J. Path. Bact.*, **64**, 252.
LITTLE, C. C.—(1920) *J. exp. Zool.*, **31**, 307.—(1941) *in* 'Biology of the Laboratory Mouse.' Philadelphia (The Blakiston Co.), p. 299.
MEDAWAR, P. B.—(1948) *Quart. J. micr. Sci.*, **89**, 187.
MITCHISON, N. A.—(1953) *Nature*, **171**, 267.
MURPHY, J. B.—(1926) 'The Lymphocyte in Resistance to Tissue Grafting, Malignant Disease and Tuberculous Infection. An Experimental Study.' New York (Rockefeller Institute for Medical Research).
STASNEY, J., CANTAROW, A., AND PASCHKIS, K. E.—(1950) *Cancer Res.*, **10**, 775.